# Health Monitoring of Metallic Structures with Electromechanical Impedance and Piezoelectric Sensors

**DOI:** 10.3390/nano9091268

**Published:** 2019-09-05

**Authors:** Jianjian Zhu, Yishou Wang, Xinlin Qing

**Affiliations:** School of Aerospace Engineering, Xiamen University, Xiamen 361002, China; zhuaero@stu.xmu.edu.cn (J.Z.); wangys@xmu.edu.cn (Y.W.)

**Keywords:** metallic structures, electromechanical impedance, piezoelectric sensors, signatures extraction, health monitoring

## Abstract

In order to monitor the health condition of structures in a more sensitive and accurate way, a novel and universal methodology called direct coupling mechanical impedance (DCMI) for characteristic signatures extraction is presented in this paper. This methodology is used to obtain DCMI signatures from measured raw signatures (RSs) with the surface-bonded piezoelectric sensors (PZT), which is developed from a pertinent electromechanical impedance (EMI) theoretical model for surface-bonded circular PZT. The proposed DCMI methodology has the advantages of simple calculation and magnifying the signatures when compared with the existing methods. Combining the extracted DCMI signatures with the root mean square deviation (RMSD) index is able to quantify the correlation between the health condition and the signatures variation more effectively. To verify the effectiveness of proposed DCMI methodology, experiments are conducted on aluminum plates and a part of fuselage in detail. The experimental results sufficiently demonstrate that the presented universal DCMI methodology possesses better sensitivity than the raw signatures when utilized for evaluating the health condition of metallic structures, including those made of metal-matrix nanomaterials.

## 1. Introduction

Fatigue, external impact and corrosion are factors that are likely to cause incipient damages inside structures, including those made of metals, metal-matrix nanomaterials, which even lead to a disastrous structural failure. Hence, discovering and diagnosing the damages on the structures made by metal or metal-matrix nanomaterials in time is an extremely important issue for primary/secondary bearing structures, especially in the aeronautic community.

The conventional technique to detect the internal damages inside structures is non-destructive testing (NDT). The typical damage detecting methods of NDT include visual inspection [1,2], ultrasonic testing (UT) [3], eddy current testing (ECT) [4], penetrant testing (PT) [5] etc. Despite the NDT techniques having been widely applied in some industrial cases, most of them are usually performed off-line [6]. To implement the on-line performance evaluation, structural health monitoring (SHM) is one of the key techniques. The definition of SHM proposed by Qing et al. [7,8] is that structural health monitoring is a method for determining the integrity of structures involving the use of multidisciplinary fields including sensors, materials, signal processing, system integration and signal interpretation. The SHM has been greatly developed and widely applied in the field of metallic, concrete and composite structures since it was firstly proposed [9,10,11,12,13,14,15]. The guided (lamb) wave and electromechanical impedance are representative active methods in SHM techniques. The lamb waves are guided by geometrical boundaries of the specimen and able to propagate long distances along the contours [16]. The waves supply information on the medium integrity along its propagation path. Thus, the lamb waves can be utilized for SHM to monitoring the internal/surface damages of metal-matrix structures or composites. Yuan and Qiu et al. [17,18,19,20] investigated the damages detection and external impact monitoring with lamb wave on metallic and composite structures using the method of imaging, signals analyzing and numerical simulation. Wu et al. [21] studied the influence of cryogenic temperature on lamb waves when used for monitoring the long-term aircraft storage tanks. Kuznetsov [22] presented the closed form analytical solution for dispersion of lamb waves in FG (functionally graded) plates. Mori et al. [23] investigated the damage localization method for plates based on the time-reversal of the mode-converted lamb waves. Hossein et al. [24] and Yan et al. [25] separately studied characteristics of wave propagation in smart structures in carbon nanotubes composite shells and nanoscale periodic layered structures. The electromechanical impedance (EMI) technique is another prospective monitoring and evaluation method for structural damages, which was first proposed by Liang et al. [26], then developed by some other researchers [27,28,29,30,31,32,33]. Ai et al. [34] applied the EMI model to detect the damages inside concrete beams. Zuo and Wongi [35,36] separately studied the crack detection in pipelines based on the EMI signals analyzing. Zhu and Qing et al. [37,38] monitored the disbond inside the composite repaired structures and honeycomb sandwich composite structures. Li et al. [39] presented an electromechanical-based analytical method to study the nonlinear dynamic behavior of corrugated graphene/piezoelectric (CGP) laminated structures. Prasath et al. [40] proposed an analytical model to predict the effective electromechanical response of fiber-reinforced composites using equivalent layered approach. Wang et al. [41] covered a systematic electromechanical transfer matrix model for a novel sandwiched type flexural piezoelectric transducer. Li and Lim separately studied the coupon corrosion and structural adhesives curing with EMI technique [42,43]. Al-Sabagh et al. monitored the damage propagation in glass fiber composites by using carbon nanofibers and analyzing conductivity’s variation, which is also a reflection of impedance to some extent [44]. Alam et al. worked on impedance model of nanowires for the applications [45]. Because the significance of metal-matrix nanocomposites has also greatly increased, particularly in several advanced manufacturing industries, the monitoring of its service performance is attracting increasing attention, such as iron-alumina and particulate reinforced metal-matrix nanocomposites. Although some researchers have studied the evaluation of metal-matrix nanocomposites with nondestructive methods [46,47,48,49,50], there is still a lot of work to do in its on-line health monitoring.

To conduct a study on the issue of on-line monitoring of metal-matrix (metals and relevant nanomaterials) structures, a universal direct coupling mechanical impedance (DCMI) methodology is presented. Then, a derivation of integrating the DCMI with the index of root mean square deviation (RMSD) is also presented to quantify the correlation between damages and signatures variation. It should be particularly pointed out that the DCMI is also applicable to the structures made of metal-matrix nanomaterials. This is because these structures usually show some similarities in mechanical behaviors with the structures made of conventional metals. Hence, the verifying experiments are only conducted on aeronautic 7075-T6 aluminum alloy plates (specimen-1 and -2) and a part of aircraft fuselage (specimen-3), which are selected as typical metallic structures in this paper. It should be pointed out that the heat treatment of aluminum alloy used in experiments undergoes solution treatment first and artificial aging second. These specimens are used for simulating different structures to verify the feasibility of DCMI methodology, respectively. The experimental results demonstrate that with the use of the proposed DCMI signatures extraction approach, more sensitive signatures and better monitoring results can be obtained when compared with the measured raw signatures.

## 2. Theoretical Methods

### 2.1. Derivation of Sensitive Component

As derived and demonstrated in our previous investigation [51], a novel expression of electromechanical admittance (reciprocal to impedance) for the surface-bonded circular piezoelectric sensors (PZT) (radius = *r*, thickness = *h*) can be written as Equation (1) in plural field. The meaning of involved variables has been enumerated in nomenclatures section.

(1) $$\bar{Y}=j\omega \cdot \bar{C}\cdot \left\{1-{\bar{k}}_{p}^{2}\left[1-\frac{2}{\bar{\varphi }}\cdot \frac{J_{1}\left(\bar{\varphi }\right)}{J_{0}\left(\bar{\varphi }\right)}\cdot \frac{{\bar{Z}}_{P,sc}}{{\bar{Z}}_{P,sc}+\xi {\bar{Z}}_{str}}\right]\right\}$$

where $\bar{C}={\bar{\varepsilon }}_{33}^{T}\pi a^{2}/h$, ${\bar{k}}_{p}^{2}=2d_{31}^{2}/\left[{\bar{s}}_{11}^{E}\cdot {\bar{\varepsilon }}_{33}^{T}\left(1-\nu \right)\right]$, $\bar{\varphi }=\bar{\kappa }\cdot a$, $c_{p}=1/\sqrt{\rho {\bar{s}}_{11}^{E}\left(1-\nu^{2}\right)}$, ${\bar{s}}_{11}^{E}=s_{11}^{E}\cdot \left(1-\eta \cdot j\right)$ and ${\bar{\varepsilon }}_{33}^{T}=\varepsilon_{33}^{T}\cdot \left(1-\delta \cdot j\right)$.

The current existing EMI analytical models ignore the influence of the adhesive layer beneath the PZT while this influence is considered in the EMI model presented in this section. Moreover, a simpler method is also proposed to compute the local mechanical impedance (LMI) of host structures. ξ in Equation (1) is an introduced variable related to the adhesive layer beneath PZT, inspired by Xu’s investigation [52]. Referring to the Bhalla’s work on the square PZT [53], the admittance model for surface-bonded circular PZT can also be broken down into a more lucid form that consists of two components (component I and component II), which takes the effect of adhesive layer into consideration, as shown in Equation (2):

(2) $$\bar{Y}=\underset{Component\;I}{\underset{︸}{j\omega \cdot \bar{C}\cdot \left(1-{\bar{k}}_{p}^{2}\right)}}+\underset{Component\;II}{\underset{︸}{j\omega \cdot \bar{C}\cdot {\bar{k}}_{p}^{2}\cdot \frac{2}{\bar{\varphi }}\cdot \frac{J_{1}\left(\bar{\varphi }\right)}{J_{0}\left(\bar{\varphi }\right)}\cdot \frac{{\bar{Z}}_{P,sc}}{{\bar{Z}}_{P}+\xi {\bar{Z}}_{str}}}}$$

According to Equation (2), the component I only depends upon the material parameters of PZT, while component II partly depends on the PZT-host structure interaction due to the ${\bar{Z}}_{P,sc}$ and ${\bar{Z}}_{str}$ only appearing in component II. Hence, it can be deemed that the component II is sensitive to damage occurrence, which is capable to be used for investigating the variation of structural LMI.

If we separately define the component I and component II as non-sensitive term and sensitive term to damages, then Equation (2) can be rewritten as Equation (3):

(3) $$\bar{Y}={\bar{Y}}_{nons}+{\bar{Y}}_{sens}$$

where ${\bar{Y}}_{nons}$ denotes the component I, ${\bar{Y}}_{sens}$ denotes the component II. The term of ${\bar{Y}}_{nons}$ can also be broken down into real and imaginary part by considering the mechanical and dielectric loss effect ($\bar{\varepsilon_{33}^{T}}=\varepsilon_{33}^{T}\left(1-j\cdot \delta \right)$ and $\bar{s_{11}^{E}}=s_{11}^{E}\left(1-j\eta \right)$), which generates Equation (4). The expressions of $G_{nons}$ and $B_{nons}$ are shown in Equation (5).

(4) $${\bar{Y}}_{nons}=G_{nons}+j\cdot B_{nons}$$

where:

(5) $$\left\{ \begin{array}{l} G_{nons}=\frac{\omega \pi a^{2}}{h}\cdot \left[\varepsilon_{33}^{T}\delta +\frac{2\eta d_{31}^{2}}{s_{11}^{E}\left(1-\nu \right)\left(1+\eta^{2}\right)}\right]\\ B_{nons}=\frac{\omega \pi a^{2}}{h}\cdot \left[\varepsilon_{33}^{T}-\frac{2d_{31}^{2}}{s_{11}^{E}\left(1-\nu \right)\left(1+\eta^{2}\right)}\right] \end{array}\right.$$

Previous investigations [54,55,56] have employed the raw conductance signatures directly for SHM application. The susceptance signatures are usually deemed redundant and unable to reflect the variation of mechanical impedance obviously [32]. However, with the use of the following processing method, the influence of ${\bar{Y}}_{nons}$ can be filtered off through Equations (6) or (7), and then, the susceptance signatures can also be used for providing helpful information about damages development:

(6) $${\bar{Y}}_{sens}=\bar{Y}-{\bar{Y}}_{nons}=\left(G+j\cdot B\right)-\left(G_{nons}+j\cdot B_{nons}\right)$$

or:

(7) $${\bar{Y}}_{sens}=\left(G-G_{nons}\right)+j\left(B-B_{nons}\right)$$

Generally, $B_{sens}$ is characterized by a large magnitude, while $G_{sens}$ displays a small magnitude because of the loss effect (δ and η). In the measured raw susceptance signatures, $B_{sens}$ usually camouflages the sensitive component. It is another reason that raw susceptance signatures are rarely used for SHM in traditional EMI technique. According to our other work [51], the proposed coupling EMI analytical model for circular PZT can be used for precisely predicting the conductance and susceptance signatures under coupling condition. The deduced conductance (real part) and susceptance (imaginary part) of sensitive component (${\bar{Y}}_{sens}$) can be determined with Equation (8):

(8) $$\left\{ \begin{array}{l} G_{sens}=G-G_{nons}\\ B_{sens}=B-B_{nons} \end{array}\right.$$

As mentioned above, despite researchers deeming that the susceptance plot cannot be used for health monitoring, after filtering the component I from the raw signatures, the $B_{sens}$ plot can also reflect the structural properties changing. The signature decomposition is able to further promote the utilization of the susceptance signatures as well as structural damages identification.

The sensitive component in Equation (2) can be expressed in the plural form as Equation (9):

(9) $${\bar{Y}}_{sens}=G_{sens}+j\cdot B_{sens}=j\omega \cdot \bar{C}\cdot \bar{k_{p}^{2}}\cdot \frac{2}{\bar{\varphi }}\cdot \frac{J_{1}\left(\bar{\varphi }\right)}{J_{0}\left(\bar{\varphi }\right)}\cdot \left(\frac{{\bar{Z}}_{P,sc}}{{\bar{Z}}_{P}+\xi {\bar{Z}}_{str}}\right)$$

### 2.2. DCMI Signature Extracting Model

As described above, a newly developed universal signatures extraction approach DCMI is developed based on the novel EMI model. The DCMI signatures are calculated with a deduced fraction including the mechanical impedance of PZT and host structure, as well as adhesive layer beneath the PZT, which is a brand-new idea for impedance/admittance signatures processing on the basis of corresponding analytical model.

Although research on extracting alleged effective impedance signatures of skeletal structures from the raw signatures has been performed by Bhalla and Soh [29], there is no similar work on the circular PZT thus far. Hence, inspired by their work, a novel DCMI signatures extraction process is deduced for surface-bonded circular PZT in this section. Theoretically, the DCMI calculation process is suitable for all types of host structures, on which the circular PZT is mounted.

Firstly, in order to write conveniently in the following process of derivation, the complex expressions of $\bar{C}=C_{r}+j\cdot C_{i}$, $\bar{k_{p}^{2}}=K_{r}+j\cdot K_{i}$, $J_{1}\left(\bar{\varphi }\right)/J_{0}\left(\bar{\varphi }\right)=J_{r}+j\cdot J_{i}$ and $\bar{\varphi }=\varphi_{r}+j\cdot \varphi_{i}$ are defined. Then, Equation (9) can be rewritten as:

(10) $$\frac{1}{2\omega }B_{sens}-j\cdot \frac{1}{2\omega }G_{sens}=\left(C_{r}+j\cdot C_{i}\right)\left(K_{r}+j\cdot K_{i}\right)\left(\frac{J_{r}+j\cdot J_{i}}{\varphi_{r}+j\cdot \varphi_{i}}\right)\left(\frac{1}{1+\xi {\bar{Z}}_{str}/{\bar{Z}}_{P,sc}}\right)$$

Secondly, symbol ${\bar{Z}}_{f}$ is defined to replace the DCMI, whose subscript *f* denotes the “fraction”. The expression of ${\bar{Z}}_{f}$ is ${\bar{Z}}_{f}=\xi {\bar{Z}}_{str}/{\bar{Z}}_{P,sc}$. Then, Equation (10) can be rewritten as Equation (11):

(11) $$\alpha_{1}+\beta_{1}\cdot j=\left(\alpha_{2}+\beta_{2}\cdot j\right)\cdot \left(\frac{1}{1+{\bar{Z}}_{f}}\right)$$

where the expressions of $\alpha_{1}$, $\beta_{1}$, $\alpha_{2}$ and $\beta_{2}$ are shown in Equation (12):

(12) $$\left\{ \begin{array}{l} \alpha_{1}=\frac{1}{2\omega }B_{sens}=\frac{1}{2\omega }\left(B-B_{nons}\right)\\ \beta_{1}=\frac{1}{2\omega }G_{sens}=\frac{1}{2\omega }\left(G-G_{nons}\right)\\ \alpha_{2}=\left[\frac{\left(-C_{i}K_{i}+C_{r}K_{r}\right)\varphi_{r}}{\varphi_{i}^{2}+\varphi_{r}^{2}}+\frac{\left(C_{r}K_{i}+C_{i}K_{r}\right)\varphi_{i}}{\varphi_{i}^{2}+\varphi_{r}^{2}}\right]J_{r}-\left[\frac{\left(C_{r}K_{i}+C_{i}K_{r}\right)\varphi_{r}}{\varphi_{i}^{2}+\varphi_{r}^{2}}-\frac{\left(-C_{i}K_{i}+C_{r}K_{r}\right)\varphi_{i}}{\varphi_{i}^{2}+\varphi_{r}^{2}}\right]J_{i}\\ \beta_{2}=\left[\frac{\left(C_{r}K_{i}+C_{i}K_{r}\right)\varphi_{r}}{\varphi_{i}^{2}+\varphi_{r}^{2}}-\frac{\left(-C_{i}K_{i}+C_{r}K_{r}\right)\varphi_{i}}{\varphi_{i}^{2}+\varphi_{r}^{2}}\right]J_{r}+\left[\frac{\left(-C_{i}K_{i}+C_{r}K_{r}\right)\varphi_{r}}{\varphi_{i}^{2}+\varphi_{r}^{2}}+\frac{\left(C_{r}K_{i}+C_{i}K_{r}\right)\varphi_{i}}{\varphi_{i}^{2}+\varphi_{r}^{2}}\right]J_{i} \end{array}\right.$$

Furthermore, defining the expression of ${\bar{Z}}_{f}=X_{f}+j\cdot Y_{f}$ and substituting it into Equation (11) yields the expression of real part ($X_{f}$) and imaginary part ($Y_{f}$), as shown in Equation (13):

(13) $$\left\{ \begin{array}{l} X_{f}=\frac{\alpha_{1}\alpha_{2}+\beta_{1}\beta_{2}}{\alpha_{1}^{2}+\beta_{1}^{2}}-1\\ Y_{f}=\frac{\alpha_{1}\beta_{2}-\alpha_{2}\beta_{1}}{\alpha_{1}^{2}+\beta_{1}^{2}} \end{array}\right.$$

With the use of Equation (13), the real part (*X_f_*) and imaginary part (*Y_f_*) are able to be determined, and the DCMI signatures can then be obtained accordingly. The main advantage of extracted DCMI signatures is that there is no need to calculate the precise values of ξ, ${\bar{Z}}_{str}$ and ${\bar{Z}}_{P,sc}$, and it only needs to calculate the specific values of these three variables, which is greatly simplified the calculation process.

In order to utilize the extracted DCMI signatures for the damage quantification more efficiently, we are to combine the extracted signatures with RMSD to assess the damage severity. The definition of RMSD is expressed in Equation (14):

(14) $$RMSD=\sqrt{\frac{\sum_{i=1}^{N}{\left[x_{h}\left(\omega_{i}\right)-x_{d}\left(\omega_{i}\right)\right]}^{2}}{\sum_{i=1}^{N}{\left[x_{h}\left(\omega_{i}\right)\right]}^{2}}}$$

where $x_{h}$ and $x_{d}$ denote the extracted DCMI signatures in health and damage condition, respectively. The notation *x* denotes the items of $X_{f}$ and $Y_{f}$, *N* is the number of measured data points and $\omega_{i}$ is the angular frequency of *i*^th^ data point [38].

## 3. Experimental Setup

### 3.1. Experiments on Aluminum Plates (Specimen-1 and -2)

In order to validate the feasibility of presented DCMI methodology and damage quantification approach, the confirmatory experiments are amply conducted in this section. The raw signatures are separately acquired via surface-mounted PZT on two aluminum plates, which are separately simulating the single damage in-situ propagation and multi-damage ex-situ development. Then, the acquired raw signatures are used for extracting DCMI signatures according to the aforementioned calculation process. The experimental setup involved in this section concentrates on investigating the correlation between the damage growth and signatures variation for metallic structures.

**Figure 1:**
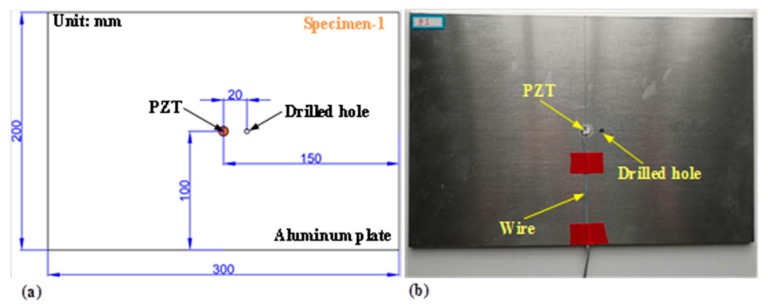
Simulated damage in-situ propagation with increasing hole’s diameter on specimen-1: (**a**) distribution of the drilled hole, (**b**) experimentally fabricated specimen.

By separately measuring the raw signals under healthy and damage conditions, any damage giving rise to structural impedance variation can be evaluated. Empirically, drilling holes on the structural surface and enlarging its diameter is an easier and more controllable work than fabricating other types of damages. Hence, the damage condition is just simulated through drilling a hole on the aluminum plate (specimen-1), whose thickness is 5 mm. The diameter of drilled hole is small enough compared with the size of aluminum plate in terms of damage area ratio to total surface area, as shown in Table 1. The distribution of the drilled hole is illustrated in Figure 1.

**Figure 2:**
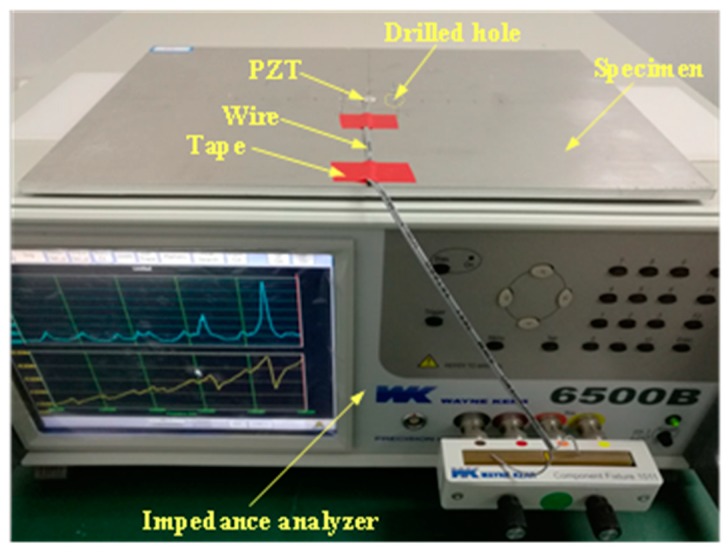
Testing platform used in experiments.

Generally, the impedance signal processing is focused on the frequency scope below 500 kHz, while a few of investigations concentrate on high frequency. Hence, a scope from 500 kHz to 2.5 MHz is selected in experiments to study the effectiveness of DCMI under the action of high frequency. The data are acquired with a precise WK-6500B impedance analyzer with a step of 5 kHz. The PZT is excited under harmonic voltage whose amplitude is 1 V. The hardware and the instrumentation used in our experiments have been shown in Figure 2. Parameters of circular PZT used in the pertinent experiments are enumerated in Table 2.

**Figure 3:**
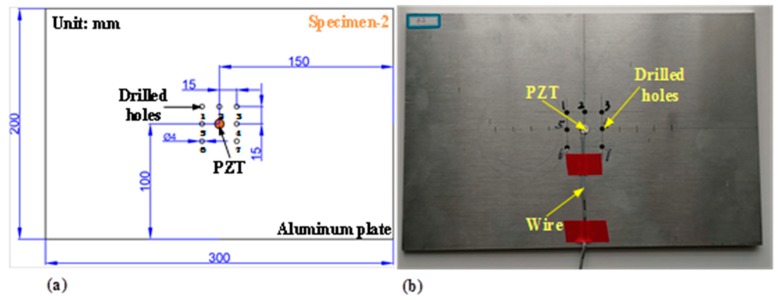
Simulated damage severity development by adding drilled holes on specimen-2: (**a**) distribution of seven drilled holes, (**b**) experimentally fabricated specimen with seven drilled holes.

For the purpose of studying the effectiveness of DCMI extraction approach in a more extensively way, another experiment was also conducted on the aluminum plate (specimen-2). Seven holes with an identical diameter of 4 mm were drilled on the specimen surface. Then the raw signatures were acquired with impedance analyzer in the states from ST-0 to ST-7, respectively. The testing specification on specimen-2 is shown in Table 3 and the distribution of seven drilled holes is illustrated in Figure 3. The development of damage severity is simulated by adding the number of drilled holes.

### 3.2. Experiment on the Real Aircraft Fuselage (Specimen-3)

**Figure 4:**
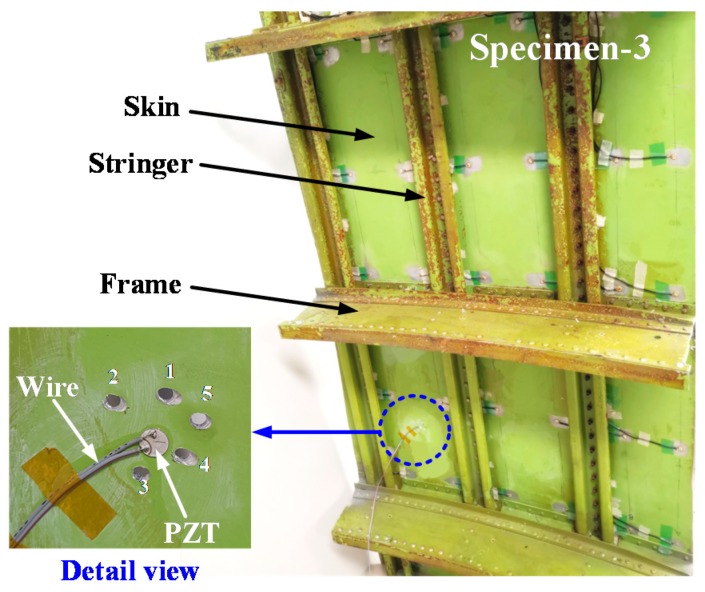
A part of real aircraft fuselage (specimen-3) used in experiment.

To demonstrate the effectiveness of DCMI methodology in practical and engineering issues, a part of the fuselage was selected to study the damage growth on a complex structure. The specimen of fuselage used in the experiment is shown in Figure 4, where the stiffened structures on the internal surface can be clearly observed. What should be pointed out is that it is more difficult to manually drill holes on the large surface than on the small aluminum plate, thus, there are inevitable deviations in the process of fabricating the damages by drilling holes on the fuselage. As can be seen in the detail view, five through-holes were created with the electric drill, whose broaches were 4 mm in diameter. The sequence of drilling the hole is also numbered in the detail view. The testing states in this experiment on fuselage are enumerated in Table 4. The frequency scope, sampling rate and excitation voltage were completely identical in specimen-1 and -2.

## 4. Results and Discussion

In this section, comparative analyses on the basis of obtained raw signatures and extracted DCMI signatures are performed. Moreover, so as to investigate the correlation between damage development and raw/extracted signatures variation, histograms of RMSD are drawn. Then, the effectiveness of presented novel DCMI extraction approach is discussed.

### 4.1. Comparative Analysis of Damage In-Situ Propagation on Specimen-1

As above-stated, the damage in-situ propagation was simulated by enlarging the diameter of the drilled hole. The raw conductance ($G_{P}$) and susceptance ($B_{P}$) signatures of circular PZT were acquired with an analyzer, and then, the corresponding DCMI signatures were extracted based on the pertinent raw signatures under each damage condition.

**Figure 5:**
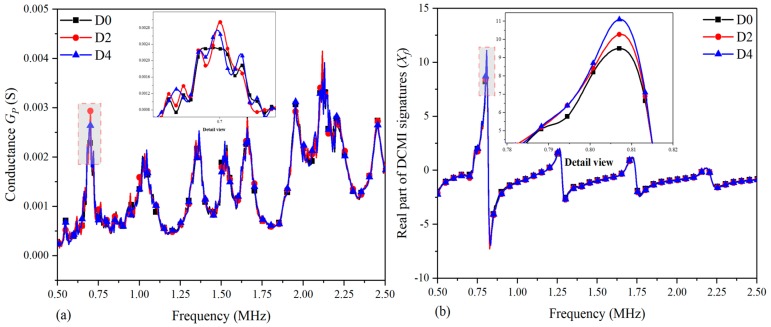
Comparative plots based on real part of signatures for specimen-1: (**a**) raw conductance signatures $G_{P}$, (**b**) extracted direct coupling mechanical impedance (DCMI) signatures $X_{f}$.

Figure 5 shows the comparative analysis between the real part of raw signatures ($G_{P}$) and DCMI signatures (*X_f_*) for specimen-1. Figure 5a shows the correlation between signatures variation and damage propagation, which is simulated with the increasing diameter of the drilled hole. Overall, according to the regularity of curves changing, it can be clearly seen that there are no obvious peaks, which is not sensitive enough to characterize the damage development. Because neither distinct amplitude variation nor obvious frequency shift is found, it is difficult to identify the damage development in terms of raw signatures. To solve this problem to a certain extent, the above-mentioned DCMI approach was adopted to extract more useful signatures from measured raw signatures.

In Figure 5b, obvious peaks are found in all the three curves (D0, D2 and D4) within 1.0 MHz. In addition, the curve becomes smoother and the difference between curves becomes more obvious, as can be seen in the detail view. With the damage growth, the amplitude of curves changed distinctly and regularly. As shown in Figure 5b, the peak frequency barely shifts, but the amplitude is obviously increased from the state of D0 to D4 in terms of detail view, which indicates that the largest amplitude is occurred in the curve of D4, while the lowest amplitude is found in the curve of D0. Apart from the first peak, there are also other obvious peaks in Figure 5b, but they are much smaller than the first peak in amplitude. Hence, when selecting the characteristic peak, only the variations at the main peak were considered in this paper. On the other hand, according to our repeated trials, there are larger differences at peak or trough tips between curves when the damage growing. That is why we selected the characteristic peak (or trough) in the experiments.

**Figure 6:**
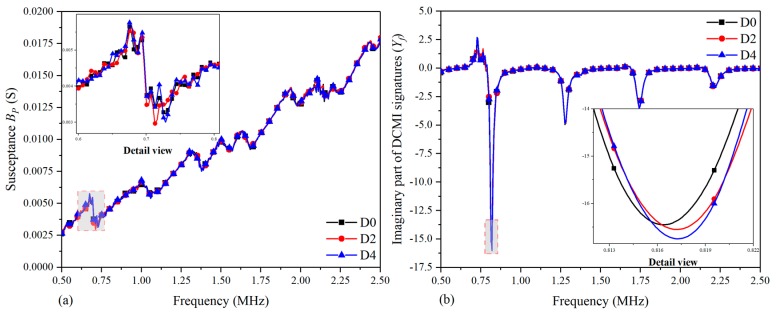
Comparative plots based on imaginary part of signatures for specimen-1: (**a**) raw susceptance signatures $B_{P}$, (**b**) extracted DCMI signatures $Y_{f}$.

Figure 6 shows the correlation between imaginary part of raw signatures ($B_{P}$) and DCMI signatures (*Y_f_*). As above-mentioned, the susceptance signatures are conventionally believed having weak interactions with the structural damage; thus, it is usually not adopted to investigate the correlation between damages and signatures variation in EMI techniques. In Figure 6a, there are curves that show the overall trend of monotonously increasing and there are also no visible peaks or frequency shift can be selected to be used for characterizing the damage variation. However, from the author’s viewpoint, the imaginary part of signatures can also provide helpful information if the proper method is applied.

By using the developed DCMI approach to extract the corresponding signatures from measured raw signatures, it can be clearly seen that there are visible peaks in Figure 6b; however, the most obvious peak exists in the frequency scope from 0.75 MHz to 1.00 MHz. The smoothness of each curve is improved and more obvious variation between each curve is found in the detailed view. In light of the detailed view in Figure 6b, the lowest amplitude is found in D4 and the highest amplitude is found in D0. In addition, the peak frequency has an obvious shift towards the right. As shown in the illustration, the highest peak frequency appears in the state of D4 while the least peak frequency appears in the state of D0. Hence, with the use of DCMI extraction approach, the susceptance signatures can also be used for investigating the damage propagation regularity, and the extracted DCMI signatures from susceptance signatures are sensitive to the damage development.

For the purpose of comparing the extracted signatures and raw signatures, as well as demonstrating the effectiveness of DCMI extraction approach comprehensively, an index of RMSD is adopted to quantify the correlation between signatures variation and damage in-situ propagation. The RMSD values are separately calculated based on the raw signatures and extracted DCMI signatures. The calculated RMSD values are shown in Table 5. Subsequently, histograms were plotted to compare the degree of signatures variation between raw signatures and extracted signatures. Moreover, it should be pointed out that the value of RMSD represents the deviation degree of damaged states from the healthy states on the grounds of Equation (14).

**Figure 7:**
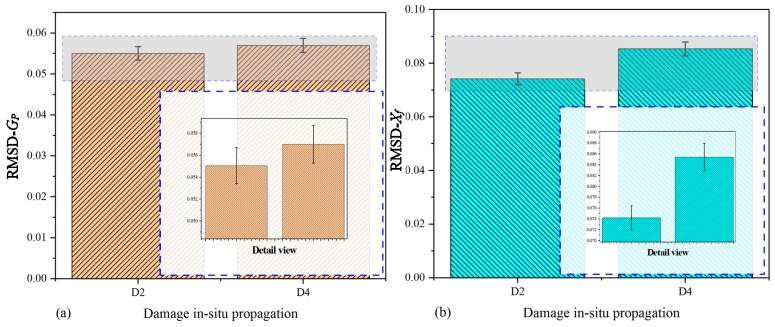
RMSD calculated based on real part of signatures for specimen-1: (**a**) raw conductance signatures $G_{P}$, (**b**) extracted DCMI signatures $X_{f}$.

Figure 7 shows the bar graph that is separately plotted on the basis of raw signatures and extracted DCMI signatures, including the detailed views. In Figure 7a, it can be clearly seen that the two bars of D2 and D4 have different heights to a certain extent, whose difference between two states can be quantitatively characterized. However, as described above, the raw signatures contain too much unhelpful information, which is adverse for RMSD calculation. As a result, it is necessary to calculate the RMSD of DCMI signatures, which are a group of more sensitive signatures that eliminate the influence of component I and only contain the influence of three items (adhesive layer, PZT and host structure), as plotted in Figure 7b. It can be clearly seen that the magnitude of RMSD calculated based on DCMI signatures is larger than that based on raw signatures, which indicates a more apparent deviation between curves of D2 and D4. The larger difference in Figure 7b indicates more excellent sensitivity of DCMI in characterizing the correlation between signatures varying and damage propagation. On the other hand, the effectiveness of combining extracted DCMI signatures with RMSD is also demonstrated.

**Figure 8:**
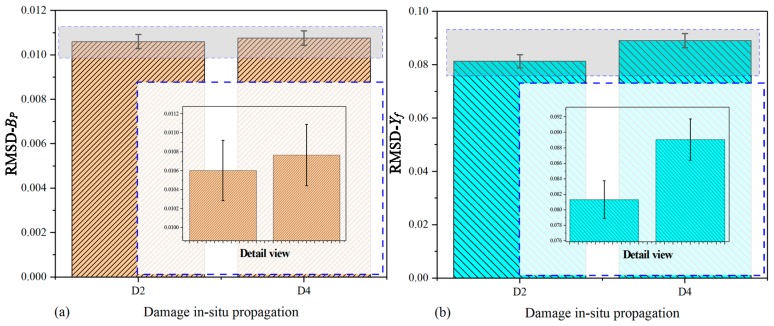
RMSD calculated based on imaginary part of signatures for specimen-1: (**a**) raw susceptance signatures $B_{P}$, (**b**) extracted DCMI signatures $Y_{f}$.

Figure 8 shows the analogous results when compared it with Figure 7. The magnitude of RMSD calculated based on raw susceptance signatures is smaller than that based on the extracted DCMI signatures, which proves the DCMI signatures is also suitable for susceptance signatures. Furthermore, the difference between two bars in Figure 8b is also more distinct than that in Figure 8a. Herein, it convincingly demonstrates that the imaginary part of extracted DCMI signatures is able to be used for quantifying the correlation between damage and signatures variation.

### 4.2. Comparative Analysis of Multi-Damage Development on Specimen-2

In order to investigate the effectiveness of DCMI approach more extensively and sufficiently, apart from the study of damage in-situ propagation on specimen-1, another experiment is also conducted on specimen-2. As stated in Section 3.1, the structural damage severity development is simulated through increasing the number of drilled holes on the aluminum plate. Seven holes were drilled successively and raw signatures were acquired in each state. Then, the pertinent DCMI signatures were extracted from the raw signatures. The sequence of drilling hole has been illustrated in Figure 3 and the damage states of specimen-2 are enumerated in Table 3.

**Figure 9:**
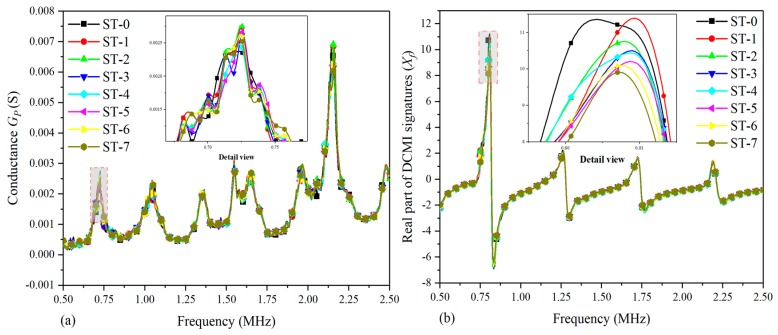
Comparative plots based on real part of signatures for specimen-2: (**a**) raw conductance signatures $G_{P}$, (**b**) extracted DCMI signatures $X_{f}$.

Figure 9 shows the comparative plots based on the real part of the raw signatures (conductance) and extracted signatures (*X_f_*). In order to study the variation in amplitude effectively, the detailed views of the main characteristic peak are plotted based on raw signatures and extracted signatures within the selected frequency scope, respectively. In Figure 9a, there are multiple peaks in the curves from ST-0 to ST-7 and there is a visible peak around the frequency point of 2.125 MHz; however, the detailed view in Figure 9a indicates that the regularity of curves variation at the concerned peak is too complicated to analyze. As a consequence, it cannot characterize the correlation between damage development and signatures variation sufficiently and effectively. Hence, the related DCMI signatures need to be extracted from the raw conductance signatures of specimen-2, as shown in Figure 9b. It can be clearly seen that an obvious peak is observed around the frequency scope from 0.75 MHz to 1.00 MHz, which can be used for quantifying the correlation between the damage and signatures variation. The magnitude of extracted DCMI signature is significantly greater than that in Figure 9a. According to the detail view of Figure 9b, the peak frequency of damage states (ST-1 to ST-6) shift towards right from the healthy state (ST-0), then the amplitude of damage states altering obviously. Therefore, the extracted DCMI signatures possessed not only the characteristics of frequency shifting, but also the obviously altering in amplitude when compared with Figure 9a. Furthermore, similar to the experimental results of specimen-1 and -2, the smoothness of each curve is improved and more obvious variation between each curve is found in terms of detail view.

**Figure 10:**
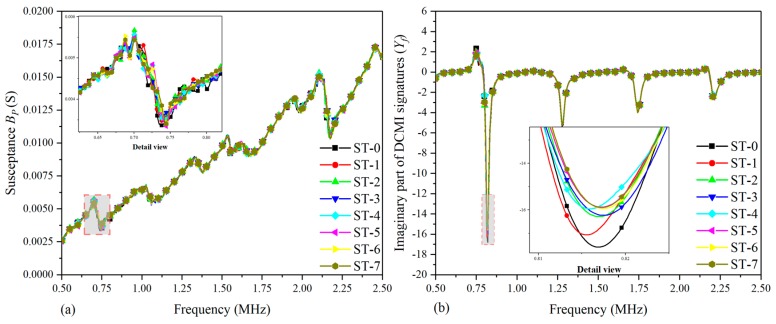
Comparative plots based on imaginary part of signatures for specimen-2: (**a**) raw susceptance signatures $B_{P}$, (**b**) extracted DCMI signatures $Y_{f}$.

Figure 10 shows the correlation between damages and signatures variation based on the imaginary part of raw signatures (susceptance) and extracted DCMI signatures (*Y_f_*). In Figure 10a, it can be clearly seen that a monotone increasing trend of curves is found and there are also no visible peaks or frequency shifts can be selected to be applied to characterizing the damage variation. Although the susceptance signatures are believed to be not very suitable for structural damage diagnosing, with appropriate signal processing approach, it can also be used for damage evaluation. With the use of the developed DCMI approach to extract the pertinent signatures from measured raw signatures, it is easy to note that there are distinct peaks in Figure 10b, but the most obvious peak is also in the frequency scope from 0.75 MHz to 1.00 MHz, which is along the negative direction of the coordinate axis. As can be seen in the detailed view in Figure 10b, the smallest amplitude is found in ST-0 and the highest amplitude is found in ST-7 and the rest of curves present gradient variation in amplitude at the first peak. In addition, the peak frequency has noticeable shift towards left with the developing of damage severity. Hence, according to the comparative results shown in Figure 10, the effectiveness of DCMI approach and extracted signatures is further demonstrated.

In order to compare the extracted signatures with raw signatures, as well as demonstrating the validity of DCMI extraction approach comprehensively, the RMSD is also adopted to quantify the correlation between signatures variation and damage severity development in this multi-damage case. Similar to the experiment performed on the specimen-1, the RMSD values are separately calculated based on the raw signatures and extracted DCMI signatures, including their real part and imaginary part. The calculated RMSD values of specimen-2 are shown in Table 6. Subsequently, corresponding histograms are plotted to study the difference between raw signatures and extracted signatures in detail.

**Figure 11:**
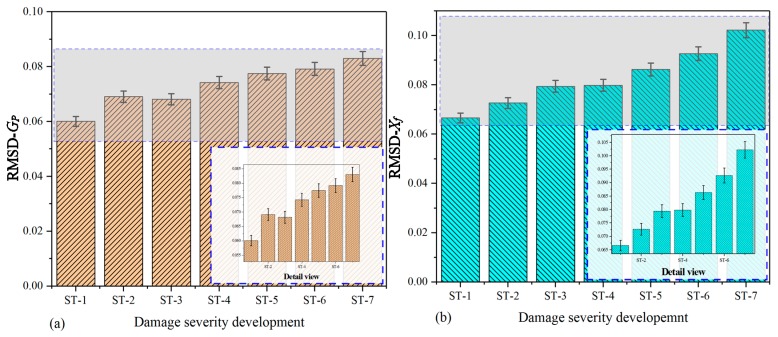
RMSD based on real part of signatures for specimen-2: (**a**) raw conductance signatures $G_{P}$, (**b**) extracted DCMI signatures $X_{f}$.

Figure 11 shows the histogram that is separately plotted on the basis of raw conductance and extracted DCMI signatures (*X_f_*). In Figure 11a, it can be clearly seen that the bars for ST-1 to ST-7 have different heights, which can be applied to quantitatively characterizing the variation among different states. Overall, the changing regularity of bars display a gradually increasing tendency. The RMSD values calculated based on raw conductance signatures are able to reflect the correlation between index and damage severity development to a certain degree, but the DCMI shows more sensitive signatures, which is directly related to the mechanical impedance of PZT, adhesive layer and the host structure, as described above. In Figure 11b, the RMSD values based on real part of DCMI signatures (*X_f_*) are calculated. More regular and apparent changing features are observed in terms of the bar’s height variation.

**Figure 12:**
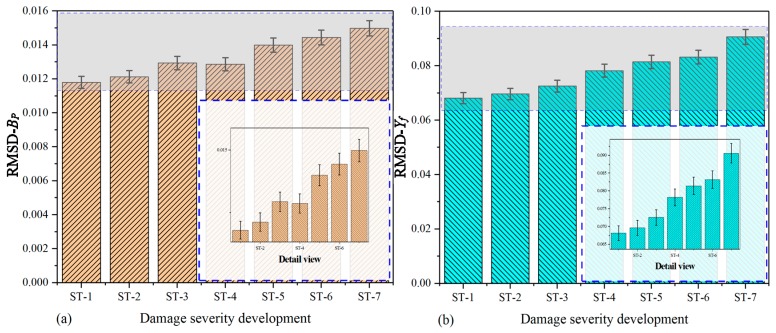
RMSD based on imaginary part of signatures for specimen-2: (**a**) raw susceptance signatures $G_{P}$, (**b**) extracted DCMI signatures $Y_{f}$.

Figure 12 shows the histograms plotted based on the imaginary part of raw signatures (susceptance) and extracted DCMI signatures (*Y_f_*). By taking a closer look at the Figure 12a,b, the magnitude of RMSD values of susceptance is much smaller than that calculated based on the imaginary part of DCMI signatures. The height variation of each bar in Figure 12a is not displaying a strictly monotone tendency. However, the variation tendency of extracted DCMI signatures presents more prominent differences among the bars. The regularity of bars changing in height is easy to be recognized and analyzed, as shown in Figure 12b.

### 4.3. Comparative Analysis of Damage Growth on Fuselage (Specimen-3)

With the setup shown in Section 3.2, an experiment was conducted on the fuselage to acquire the raw conductance ($G_{S}$) and susceptance ($B_{S}$) signatures with an impedance analyzer. Then, the RMSD values are calculated and the pertinent curve charts, as well as histograms, were plotted.

**Figure 13:**
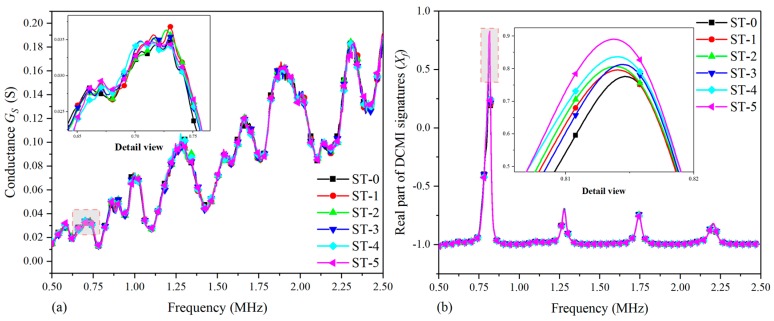
Comparative plots based on real part of signatures for fuselage: (**a**) raw conductance signatures $G_{s}$, (**b**) extracted DCMI signatures $X_{f}$.

Figure 13 shows the comparative plots based on raw conductance signatures and extracted DCMI signatures. According to Figure 13a, it can be clearly seen that there are no obvious regularity can be used for analyzing the correlation between damage growth and amplitude variation. On the contrary, with the use of DCMI methodology, very distinct characteristic peaks can be found in the same frequency scope, which is suitable to be used for investigating the influence of damage growth on host structure, as shown in Figure 13b.

In order to take a closer look at the first peak, a detail view is plotted both in Figure 13a,b. It should be pointed out that the detail view in Figure 13a is too complex to analyze, while the more clear regularity can be found in amplitude and frequency variation in Figure 13b. The largest amplitude appears in the curve of ST-5 and the least amplitude in the first peak appears in the curve of ST-0. The rest of curves from ST-1 to ST-4 change gradually in their amplitudes as well as slightly shift in peak frequency. Apart from the main characteristic peak in Figure 13b, another three lower peaks are found, but they are much smaller in amplitude when compared with the first peak. Hence, they are not adopted to be used for studying the damage-signature interaction.

**Figure 14:**
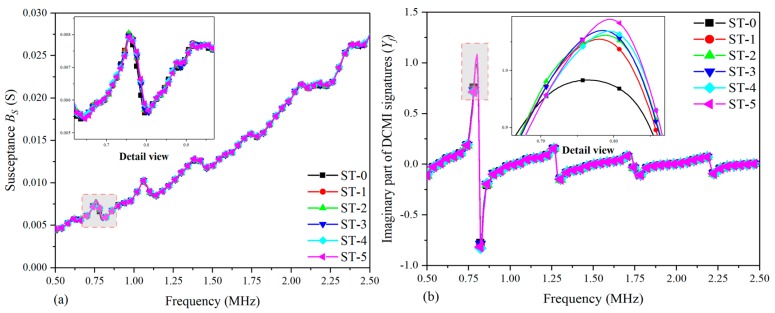
Comparative plots based on imaginary part of signatures for fuselage: (**a**) raw susceptance signatures $B_{s}$, (**b**) imaginary part of DCMI signatures $Y_{f}$.

Figure 14 shows the comparative plots based on raw imaginary signatures and extracted DCMI signatures. It can be clearly seen that there shows monotonous variation tendency in Figure 14a, but there are no obvious peaks to be used for analyzing the regularity of signatures variation. However, the raw susceptance signatures are processed with DCMI methodology and very distinct characteristic peaks are extracted in the selected frequency scope, which is appropriate to be used for studying the impact of damage propagation on host structure, as shown in Figure 14b.

For the purpose of taking a closer look at the first peak, a detail view is also plotted in Figure 14a,b. Note that the detail view in Figure 14a contains differences too small to be analyzed, while a more clear regularity can be found in the amplitude and frequency variation in Figure 14b, although not as obvious as that in the real part of DCMI signatures. The largest amplitude at the first peak appears in the curve of ST-5 and the least amplitude appears in the curve of ST-0. The rest of the curves from ST-1 to ST-4 vary slowly in their amplitudes as well as slightly shift in peak frequency. According to the results shown in Figure 14, it is easy to know that the susceptance signatures are not as sensitive as the conductance signatures obtained from fuselage with the impedance analyzer; however, using the DCMI method, the susceptance signatures can also provide supplementary information for investigations on damage-signature correlation.

**Figure 15:**
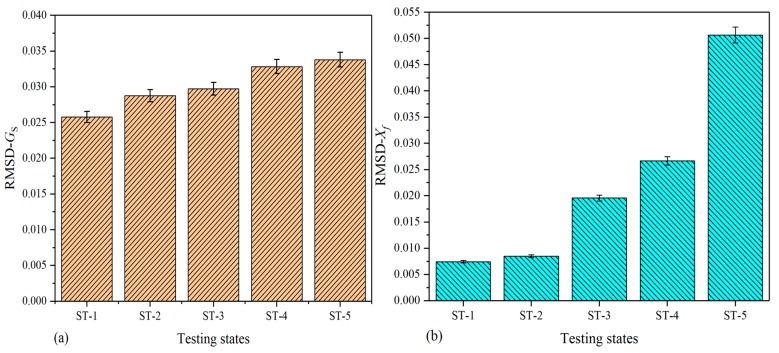
RMSD based on real part of signatures for fuselage: (**a**) raw conductance signatures $G_{s}$, (**b**) extracted DCMI signatures $X_{f}$.

**Figure 16:**
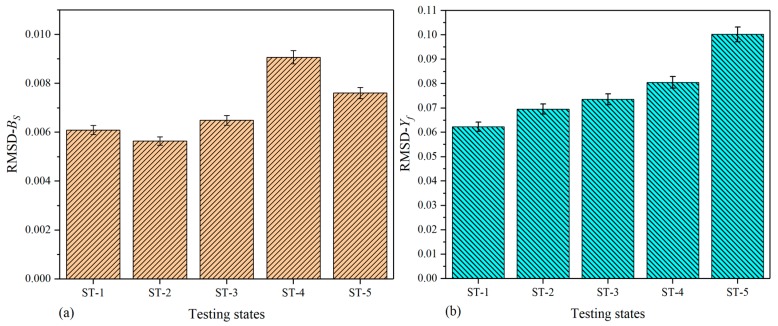
RMSD based on imaginary part of signatures for fuselage: (**a**) raw susceptance signatures $B_{s}$, (**b**) extracted DCMI signatures $Y_{f}$.

To investigate the signatures changing with damage growth, the RMSD values were also calculated in terms of Equation (14); however, the RMSD values of extracted DCMI signatures are slightly different from that of specimen-1 and -2 because of the signatures acquired from the fuselage being too complex. The main difference lies in the number of data point used for RMSD calculation. For the purpose of reducing data processing scale, a novel method was developed. All of peak points in Figure 13b and Figure 14b were separately selected based on the concern that the curve peaks have the largest variation not only in amplitude but also in frequency shift. Contrarily, the rest of data points are not utilized with the use of this method. Thus, the scale of data processing can be greatly reduced and more obvious variation can be obtained. On the other hand, because the comparative results shown in Figure 15 and Figure 16 are clear enough, there is no need to illustrate detail views in these two figures.

Figure 15 shows the histograms of RMSD calculated on the basis of $G_{s}$ and $X_{f}$ (real part of the extracted DCMI signatures). In Figure 15a, it can be clearly seen that with the growth of damage, the height of RMSD bars also increase gradually. The maximum RMSD value occurs in the state of ST-5, while the smallest value occurs in the state of ST-1. In Figure 15b, larger differences among bars are observed when compared with that plotted based on $G_{s}$ signatures.

Figure 16 shows the histograms of RMSD calculated on the basis of $B_{s}$ (raw susceptance in serial measurement mode) and $Y_{f}$ (imaginary part of extracted DCMI signatures). In Figure 16a, it can be clearly seen that with the growth of damage, the height of RMSD bars changes apparently, but there are no obvious or monotonously changing regularity. In this case, the largest bar in RMSD value occurs in the state of ST-4 while the least bar occurs in the state of ST-2. It is likely to be caused by the non-sensitivity of susceptance signatures to damage growth, but with the use of DCMI methodology, as shown in Figure 16b, much larger differences among bars are observed and the monotonous regularity in the height of bars is more obvious when compared it with that plotted based on $B_{s}$ signatures. In addition, the magnitude of RMSD in Figure 16b becomes larger than that in Figure 16a on account of the fractional expression of DCMI methodology.

In this section, a DCMI extraction approach was used in experiments to obtain more sensitive signatures to study the correlation between damages growth and signatures changing. By plotting the curves and comparatively analyzing their varying regularity, it can easily be found that the smoothness of each curve is improved. Obvious variations between each curve are found in terms of detail view, as well as the differences between RMSD bars. Hence, the effectiveness of the DCMI approach is convincingly demonstrated and it has potential application in the community of EMI-based structural health monitoring.

## 5. Conclusions

In this paper, a universal DCMI signatures extraction approach is developed from a novel EMI model to monitor the health condition of metallic structures with piezoelectric sensors. Theoretically, any solid body possesses mechanical impedance; thus, the methodology is apparently suitable for structures composed of metal, metal-matrix nanomaterials and composites, the impedance of which can be measured with surface-bonded PZT. With the use of DCMI method, some issues of health monitoring are likely to be solved in an effective way, which has practical significance in engineering structures. The developed extraction approach is used to obtain a series of more sensitive signatures to investigate the influence of damages on structures. Three experiments, damage in-situ and ex-situ development on aluminum plates, as well as aircraft fuselage, are conducted to study the effectiveness of the methodology, respectively.

Furthermore, in order to quantify the correlation between damages and EMI signatures variation, the statistical index RMSD is adopted. Through combining the DCMI signatures with RMSD, the influence on structures caused by damages in-situ propagation or ex-situ development are capable of being well quantified. Hence, the feasibility and effectiveness of DCMI methodology can then be convincingly demonstrated. Based on the comprehensive and detailed comparison, the following remarks can be made:

(1) As long as the PZT is surface-bonded on the structures, the DCMI methodology can be used for monitoring the damage propagation on simple and complex structures, which are made of metals, metal-matrix nanomaterials and composites.
(2) More obvious peaks can be extracted from the raw signatures with the use of DCMI methodology, which may have potential applications in the SHM community in the future.
(3) The correlation between damages growing and signatures variation can be well quantified via combing DCMI signatures with the RMSD index.
(4) The DCMI methodology can extract more sensitive signatures from measured raw signatures in a simpler way.
(5) The susceptance signatures can also be used for assessing the regularity of damage changing after extracting signatures with the DCMI.

## Figures and Tables

**Table 1:** Testing specifications of specimen-1.

Testing Specifications	Diameter of Drilled Hole	Damage Area Ratio (%)
D0	—	—
D2	2 mm	5.236 × 10^−3^
D4	4 mm	0.021

**Table 2:** Typical parameters of circular piezoelectric sensors (PZT).

Physical Parameters	Names	Values
$S_{11}^{E}$(10^−12^*m*^2^/*N*)	Compliance coefficient at constant electric field	16.43
η	Mechanical loss factor	0.025
$\varepsilon_{33}^{T}/\varepsilon_{0}$	Relative permittivity	1920
*δ*	Dielectric loss factor	0.01
*d*_31_(×10^−12^*C*/*N*)	Piezoelectric strain coefficient	−200
ν	Poisson’s ratio	0.32
*ρ* (*kg/m*^2^)	Density	7750
*h* (10^−3^ *m*)	Thickness	0.5
*a* (10^−3^ *m*)	Radius	4.2

**Table 3:** Simulated multi-damage severity development by adding the drilled holes on specimen-2.

Testing States on Specimen-2	Number of Drilled Hole (mm)
ST-0	0 (Baseline)
ST-1	1
ST-2	2
ST-3	3
ST-4	4
ST-5	5
ST-6	6
ST-7	7

**Table 4:** Simulated damage growth on the aircraft fuselage (specimen-3).

Testing States on Fuselage	Number of Drilled Hole (mm)
ST-0	0 (Baseline)
ST-1	1
ST-2	2
ST-3	3
ST-4	4
ST-5	5

**Table 5:** Root mean square deviation (RMSD) based on raw signatures and DCMI signatures of specimen-1.

States	Raw Signatures		DCMI Signatures	
	*G_P_*	*B_P_*	*X_f_*	*Y_f_*
D2	0.05505	0.01060	0.07423	0.08133
D4	0.05700	0.01077	0.08542	0.08907

**Table 6:** RMSD based on raw signatures and DCMI signatures of specimen-2.

States	Raw Signatures		DCMI Signatures	
	*G_P_*	*B_P_*	*X_f_*	*Y_f_*
ST-1	0.06008	0.01180	0.06659	0.06815
ST-2	0.06907	0.01212	0.07267	0.06965
ST-3	0.06816	0.01294	0.07942	0.07258
ST-4	0.07423	0.01287	0.07984	0.07821
ST-5	0.07747	0.01400	0.08631	0.08144
ST-6	0.07918	0.01444	0.09268	0.08319
ST-7	0.08300	0.01498	0.10221	0.09061

## Nomenclatures

j	Complex symbol
ω	Angular frequency (rad/s)
C	Capacitance symbol (F)
$\varepsilon_{33}^{T}$	Dielectric constant (F/m)
a	Radius of circular PZT (m)
h	Thickness of circular PZT (m)
$k_{p}$	Coupling coefficient
$d_{31}$	Piezoelectric strain constant (×10^−12^ C/N)
$s_{11}^{E}$	Compliance coefficient (m^2^/N)
ν	Poisson ratio
κ	Wavenumber (m^−1^)
$c_{p}$	Phase velocity (m/s)
ρ	Density (kg/m)
η	Mechanical loss factor
δ	Dielectric loss factor
φ	Variable of Bessel function
$Z_{P,sc}$	Mechanical impedance of PZT under short-circuited condition (Ω)
$Z_{str}$	Mechanical impedance of host structure (Ω)
ξ	Symbol related to adhesive layer beneath PZT
$Y_{nons}$	Non-sensitive component in admittance model (S)
$Y_{sens}$	Sensitive component in admittance model (S)
Y	Admittance (S)
$G_{p}$	Raw conductance in parallel measurement mode (S)
$G_{s}$	Raw conductance in serial measurement mode (S)
$B_{p}$	Raw susceptance in parallel measurement mode (S)
$B_{s}$	Raw susceptance in serial measurement mode (S)
$C_{r}$	Real part of complex capacitance (F)
$C_{i}$	Imaginary part of complex capacitance (F)
$K_{r}$	Real part of complex wavenumber (m^−1^)
$K_{i}$	Imaginary part of complex wavenumber (m^−1^)
$J_{1}\left(\varphi \right)$	1-order Bessel function of the first kind
$J_{0}\left(\varphi \right)$	0-order Bessel function of the first kind
$J_{r}$	Real part of complex fraction of Bessel function
$J_{i}$	Imaginary part of complex fraction of Bessel function
$\varphi_{r}$	Real part of the variable in Bessel function
$\varphi_{i}$	Imaginary part of the variable in Bessel function
$Z_{f}$	Modulus of DCMI signatures
$X_{f}$	Real part of DCMI signatures
$Y_{f}$	Imaginary part of DCMI signatures
